# Host feeding patterns and preference of *Anopheles minimus* (Diptera: Culicidae) in a malaria endemic area of western Thailand: baseline site description

**DOI:** 10.1186/1756-3305-5-114

**Published:** 2012-06-07

**Authors:** Rungarun Tisgratog, Chatchai Tananchai, Waraporn Juntarajumnong, Siripun Tuntakom, Michael J Bangs, Vincent Corbel, Theeraphap Chareonviriyaphap

**Affiliations:** 1 Department of Entomology, Faculty of Agriculture, Kasetsart University, Bangkok, 10900, Thailand; 2 Department of Entomology, Faculty of Agriculture, Kasetsart University, Kamphaengsaen, Nakhon Pathom, 73140, Thailand; 3 Public Health & Malaria Control Department, Jl. Kertajasa, Kuala Kencana, Papua, 99920, Indonesia; 4 Institut de Recherche pour le Développement (IRD), Maladies Infectieuses et Vecteurs, Ecologie, Génétique, Evolution et Contrôle (IRD 224-CNRS 5290 UM1-UM2), Montpellier Cedex 5, France

**Keywords:** *Anopheles minimus*, Seasonal abundance, Blood feeding behavior, Host preference, Malaria, Thailand

## Abstract

**Background:**

Host feeding patterns of *Anopheles minimus* in relation to ambient environmental conditions were observed during a 2-year period at Tum Sua Village, located in Mae Sot District, Tak Province, in western Thailand, where *An. minimus* is found in abundance and regarded as the most predominant malaria vector species. Detailed information on mosquito behavior is important for understanding the epidemiology of disease transmission and developing more effective and efficient vector control methods.

**Methods:**

Adult mosquitoes were collected every 2 months for two consecutive nights from 1800 to 0600 hrs. Three collection methods were used; indoor human-landing collections (HLC), outdoor HLC, and outdoor cattle-bait collections (CBC).

**Results:**

A total of 7,663 female *Anopheles* mosquitoes were collected of which 5,392 were identified as members of 3 different species complexes, the most prevalent being *Anopheles minimus* complex (50.36%), followed by *Anopheles maculatus* complex (19.68%) and *Anopheles dirus* complex (0.33%). *An. minimus* s.s. comprised virtually all (> 99.8 percent) of Minimus Complex species captured. Blood feeding behavior of *An. minimus* was more pronounced during the second half of the evening, showing a slight preference to blood feed outdoors (~60%) versus inside structures. Significantly (*P* < 0.0001) more *An. minimus* were collected from human-baited methods compared with a tethered cow, indicating a more anthropophilic feeding behavior. Although a significant difference in total number of mosquitoes from the HLC was recorded between the first and second year, the mean biting frequency over the course of the evening hours remained similar.

**Conclusions:**

The Human landing activity of *An. minimus* in Tum Sua Village showed a stronger preference/attraction for humans compared to a cow-baited collection method. This study supports the incrimination of *An. minimus* as the primary malaria vector in the area. A better understanding of mosquito behavior related to host preference, and the temporal and spatial blood feeding activity will help facilitate the design of vector control strategies and effectiveness of vector control management programs in Thailand.

## Background

In recent years, approximately two-thirds of all recorded malaria cases in Thailand have been found along the international border of Thailand-Myanmar [1,2]. Between 2008 and 2010, averages of 7,377 (± 1,662) malaria cases were recorded annually in Mae Sot District [3]. The malaria epidemiology and persistence of disease transmission in the area has been primarily associated with small-scale agricultural activity, both occupationally-related and uncontrolled tribal population movements, and periodic political/civil unrest producing displaced populations in transient conditions more conducive for malaria transmission [1]. The site (Tum Sua Village, Mae Sot District) utilized in this study is considered a perennial malaria transmission area. Several important vectors of malaria are prevalent in the area, including members of the *Anopheles minimus* complex [4], one of the most important malaria vectors in rural forested and scrub areas of western Thailand [1,5,6].

At least two genetically distinct sibling species, *An. minimus* and *Anopheles harrisoni*, belong to the Minimus Complex [7]. These two species are difficult to identify accurately based on morphological characters alone [8,9]. In Thailand, *An. minimus* is regarded as the most predominant malaria vector species and is found throughout the country [10,11], whereas *An. harrisoni* is restricted along the western Thailand-Myanmar border, particularly in Kanchanaburi Province [7,11-13].

Allozyme analysis, once regarded as the gold standard to differentiate members in the Minimus Complex [10] has been replaced by several molecular, nucleic acid based approaches for more accurately separating different species within this complex. Two PCR-based techniques, an allele-specific amplification (ASA) and a single-strain conformation polymorphism (SSCP) assay have been developed for distinguishing members of the Minimus Subgroup and other closely related species [14]. Both multiplex format and a PCR-restriction fragment length polymorphism (RFLP) method were also developed to distinguish species within the Minimus Complex and other related species, namely *An. minimusAn. harrisoni, Anopheles aconitusAnopheles pampanai* and *Anopheles varuna*, all species are found in Thailand [7,15,16]. A single multiplex PCR assay, using sequence characterized amplified region (SCAR) markers derived from individual random amplified polymorphic DNA is also able to differentiate between *An. minimus* and *An. harrisoni*, and their hybrids, as well as the 3 closely related species within the Minimus Subgroup [17,18].

Details of mosquito biology, especially host feeding activity and preference of individual species within the complex is essential to identify their respective roles in disease transmission and assist vector control operations to apply the most appropriate strategy of vector control management. Other observations on biting cycles and host preferences of *An. minimus* have been made in Thailand [19-24]; however, nearly all ecological and behavioral studies to date have been based on populations identified by morphological traits alone. More recently, the seasonal abundance of *An. minimus* and *An. harrisoni* adults in Kanchanaburi Province were identified using accurate molecular methods [11]. Unfortunately, the low number of *An. minimus* collected in that study area, predominated by *An. harrisoni*, resulted in no conclusive information on trophic behavior and seasonal abundance for this species. Using a PCR-based molecular method to ensure species-specific identification, we described the blood feeding/biting behavior, host preference and seasonal abundance of *An. minimus* over a two-year period in Tum Sua Village.

## Methods

### Study site

*Anopheles minimus* populations were collected from the Tum Sua Village (16^o^4 ^o^ N, 98^o^41^o^E), in Mae Sot District of western Thailand. To the west, the border with Myanmar is separated by mountain ranges and the Moei River. Approximately 80 percent of the study site is surrounded by fruit orchards and other agricultural undertakings, while the remaining periphery of the village is composed of intact, sparsely populated, native forest on the west. A 2 meter -wide perennial flowing stream transects the village proper and is bordered by a variety of permanent riparian vegetation along its margins.

### Collection methods

Adult mosquito collections were conducted every 2 months for two consecutive nights from 1800 to 0600 hrs, between November 2008 and September 2010. Three collection methods were used; indoor human-landing collections (HLC), outdoor HLC and outdoor cattle-bait collections (CBC). Indoor HLC were conducted in an insecticide-free house. The indoor and outdoor HLC collectors were separated into 2 teams of 4 collectors each, each team working 6 hr evening shifts. While 2 collectors captured mosquitoes inside the house the other 2 remained stationed outside at a distance of approximately 50 m from the same house. The first team worked between 1800 and 2400 hr, followed by the second team beginning at midnight until 0600 hr. Teams were rotated between the first and second halves of the evening on the second collection night to mitigate potential collector bias. Teams also exchanged positions between indoor and outdoor collections each alternate night. Indoor and outdoor HLC took place for 45 min each hour. Outdoor CBC was conducted by a separate team of two collectors, for 15 min each hour following the methods of Sungvornyothin *et al.*[25]. The CBC involved placing a single adult cow under a clean (untreated) cotton bed net measuring 3.6 m x 3.3 m x 2.0 m (L:W:H) with the net suspended 30 cm above the ground level to allow mosquitoes access inside. The net trap was placed approximately 50 m from the nearest HLC and at equal distances from the perimeter tree line to avoid potential bias in attracting mosquitoes. The cow was exposed to mosquitoes entering the net uninterrupted for 45 min each hour. All mosquitoes, either resting inside the net or on the cow at time of collection, were captured using a mouth aspirator.

All collected mosquitoes were held in plastic holding cups and labeled by hour, location and collector name. Each cup contained cotton soaked with 10% sugar solution for sustenance. Cups were transported back to project site laboratory each hour for initial morphological identification. All *Anopheles* specimens were identified to species following Rattanarithikul *et al.*[8]. Environmental parameters were recorded each hour of collection by teams, using a manual thermo-hygrometer (BARICO GmbH, Villingen-Schwenningen, Germany). Rainfall data were obtained from the local meteorological station, approximately 18 km from the study area. Formal animal/human use approval for this research was granted by the Ethical Research Committee, Kasetsart University Research and Development Institute (KURDI), Kasetsart University, Thailand (KURDI-1/2543- 1421457).

### Molecular identification

A multiplex Allele Specific Polymerase Chain Reaction (AS-PCR) procedure was performed for molecular identification of individual adult *Anopheles* initially identified morphologically as *An. minimus* complex species**.** DNA extraction followed the protocol of Linton *et al.*[26] and AS-PCR assay by Garros *et al.*[16] to confirm the species identification.

### PCR amplification of ITS-2 region

Following DNA extraction from individual adult mosquitoes, whole or partial specimens (e.g., legs and wings), isolated DNA was subjected to sequential PCR procedures. The PCR mixture contained 17.75 μl ultrapure distilled water, 2.5 μl of 10X reaction buffer, 10 mM of each dNTP, 10 μM of primer, 0.5 units of *Taq* DNA polymerase and 0.5 μl of DNA template. After an initial denaturation step at 94 °C for two min, 40 cycles were programmed as follows: 94 °C for 30 sec, 54 °C for 30 sec, 72 °C for 40 sec, and a final extension step at 72 °C for five min. Products were visualized using electrophoresis on a 2% agarose gel. Primer names, sequences and sizes of the PCR products are shown in Table 1. The internal transcribed spacer 2A (ITS-2A) is the universal primer that binds to the same position on the ITS rDNA for 10 closely related species (including 5 anopheline species in the Funestus Group present in tropical Africa only), while the five specific primer (*Pam* to *Mia*) PCR reactions bind at different locations on the ITS-2 sequence of each corresponding species.

**Table 1:** **Primers, sequences, and sizes of PCR products used in the molecular identification of*Anopheles minimus*complex species present in Thailand**

**Species**	**Primer name**	**Sequence (5’ to 3’)**	**Size of the product (bp)**	**Tm (^o^C)**
Universal forward primer	ITS2	TGT GAA CTG CAG GAC ACA T		54.5
*Anopheles pampanai*	PAM	TGT ACA TCG GCC GGG GTA	90	56.0
*Anopheles aconitus*	ACO	ACA GCG TGT ACG TCC AGT	200	58.2
*Anopheles harrisoni*	MIC	GTT CAT TCA GCA ACA TCA GT	180	53.2
*Anopheles varuna*	VAR	TTG ACC ACT TTC GAC GCA	260	53.7
*Anopheles minimus*	MIA	CCC GTG CGA CTT GAC GA	310	57.6

*The internal transcribed spacer 2 (ITS2A) is the universal primer that binds to the same position on the ITS2 DNA for all 5 species, while the specific primers (PAM to MIA) bind at different places on the ITS2 DNA of the corresponding species. bp = number base pairs; Tm = melting temperature.

### Data analysis

Three key factors were chosen for statistical analysis, 1) Climatic seasons - wet period (June to October), dry period (November to February) and a hot period (March to May), 2) Collection periods - early evening (1800–2100 hr), late night (2100–2400 hr), pre-dawn (2400–0300 hr) and dawn (0300–0600 hr) and 3) Collection types - indoor HLC, outdoor HLC and cow-baited captures. The nocturnal biting activity of *An. minimus* was analyzed by mean number of landing mosquitoes captured per human each hour separated by indoor and outdoor collections and by mean number of mosquitoes captured from the CBC per hour. Comparisons of landing capture data were analyzed by a three-way analysis of variance (ANOVA), with year of collection as the blocked factor. Differences among collection groups were compared using Duncan’s multiple-range test [27]. The level of significance was set at 5% (*P*-value < 0.05). All data were analyzed using a SAS statistical package (SAS Release 6.01, SAS Institute, Cary, NC, USA).

Correlation analysis was used to examine the relationship and estimate differences between number of mosquitoes and the independent environmental variables of ambient temperature and relative humidity by hour of collection. Correlation coefficients (*r*) between number of mosquitoes and hourly means for indoor temperature, outdoor temperature, and indoor humidity were based on the *H*_*0*_*: r* = 0*; H*_*1*_*: r* ≠ 0, *r* > 0, *r* < 0. The discriminating level for significance for all correlation tests was set at 5% (*P* < 0.05). Correlation analyses were performed using a SPSS statistical program (SPSS version 15.0 Inc. Chicago, IL, U.S.A.).

## Results

Results of adult anopheline collections performed from November 2008 to September 2010, a total of 24 all-night collections, with matching temporal ambient air temperature, humidity and rainfall data are provided in Tables 2,3,4,5 and 6. From a total of 7,663 anopheline species collected from Tum Sua Village, 5,392 (70.37%) were members within one of 3 species complexes, representing the *An. minimus* (50.36%), *An. maculatus* (19.68%) and *An. dirus* complexes (0.33%), respectively. The remaining anopheline mosquitoes (29.63%, n = 2,271) were regarded as non-malaria vectors (Table 2).

**Table 2:** **Human-landing collections of adult*Anopheles*species every 2 months from Tum Sua Village, western Thailand between November 2008 and September 2010**

**Year**	**Month**	***An. minimus***	An. dirus*	An. maculatus*	**Other *Anopheles* spp.†**
One	Nov’08	608	1	84	202
	Jan’09	190	-	54	58
	Mar’09	272	-	50	90
	May’09	366	4	81	205
	Jul’09	30	3	9	269
	Sep’09	6	8	9	123
Two	Nov’09	117	-	62	21
	Jan’10	258	1	38	82
	Mar’10	429	-	23	20
	May’10	995	-	992	66
	Jul’10	303	-	84	781
	Sep’10	285	8	22	336
	Total	3,859	25	1,508	2,271

* Specific species in complex not differentiated by molecular methods.

† Other species: *Anopheles aconitus*, *Anopheles argyropus, Anopheles barbirostris, Anopheles jamesii, Anopheles karwari, Anopheles kochi, and Anopheles peditaeniatus.*

**Table 3:** **Human-landing rates (mosquitoes/person/night) of adult*An. minimus*collected every 2 months from Tum Sua Village, western Thailand between November 2008 and September 2010**

**Year**	**Month**	**Indoor HLC rate per person/-night** **Mean(SE)**	**Outdoor HLC rate per person/-night** **Mean(SE)**
One	Nov’08	41(0.60)	42.5(0.33)
	Jan’09	14.5(0.22)	11.5(0.23)
	Mar’09	16(0.31)	32.5(0.40)
	May’09	25.75(0.40)	45.25(0.70)
	Jul’09	3.75(0.09)	0(0)
	Sep’09	0.75(0.03)	0.25(0.02)
Two	Nov’09	15.75(0.30)	8(0.16)
	Jan’10	31.5(0.40)	18.25(0.27)
	Mar’10	19.25(0.30)	57.25(0.82)
	May’10	62.25(1.19)	164.5(2.31)
	Jul’10	37.25(0.69)	35.25(0.55)
	Sep’10	31.5(0.58)	31.25(0.34)
Mean landing rate per person/night:		31.07	

**Table 4:** **Total numbers of*Anopheles minimus*captured by location and host matched with contemporaneous environmental parameters from Tum Sua Village, Mae Sot District, western Thailand**

	**Year 1**								**Year 2**								**Total No. of mosquitoes**
**Month**	***An. minimus***			**T (°C)^1^**		**RH (%)^2^**		**R^3^**	***An. minimus***			**T (°C)^1^**		**RH (%)^2^**		**R^3^**	
	**Indoor**	**Outdoor**	**Cow**	**Indoor**	**Outdoor**	**Indoor**	**Outdoor**	**Total**	**Indoor**	**Outdoor**	**Cow**	**Indoor**	**Outdoor**	**Indoor**	**Outdoor**	**Total**	
Nov	164	170	274	19.9	19.6	77.8	76.6	59.9	63	32	22	18.80	17.9	90.15	91.85	0	725
Dec†	-	-	-	-	-	-	-	10.4	-	-	-	-	-	-	-	0	-
Jan	58	46	86	21.55	21.6	79.9	71.55	0	126	73	59	18.30	19	88.8	89.25	11.5	448
Feb†	-	-	-	-	-	-	-	0	-	-	-	-	-	-	-	0	-
Mar	64	130	78	23.90	25.6	64.2	74.85	26.8	77	229	123	23.6	23.6	82.8	84.6	5.4	701
Apr†	-	-	-	-	-	-	-	78	-	-	-	-	-	-	-	0	-
May	103	181	82	28.85	26.6	76.45	66.35	155.6	249	658	88	23.8	23.85	93.65	92	104.1	1,361
Jun†	-	-	-	-	-	-	-	418.3	-	-	-	-	-	-	-	87.9	-
July	15	0	15	31.1	23.2	77.5	70.7	482.5	149	141	13	25.35	23.95	92.45	93.3	281.4	333
Aug†	-	-	-	-	-	-	-	272	-	-	-	-	-	-	-	368	-
Sep	3	1	2	24.25	24.5	77.4	79.95	213.9	126	125	34	18.7	23.7	94.4	97.25	182.9	291
Total	407	528	537	-	-	-	-	1,717.4	790	1,258	339	-	-	-	-	1,041.2	3,859

^1^ T : Mean ambient temperature.

^2^RH: Mean percent relative humidity (%).

^3^R : Total Rainfall (mm).

† : Non-collection months.

**Table 5:** **Three-way ANOVA of total number of landing*An. minimus*by hour, season (dry, hot, and wet), collection method (indoor and outdoor human bait, and outdoor cattle bait) and time interval (early evening, late evening, pre-dawn, and dawn) as discriminating factors**

**Source**	**df**	**Sum of squares**	**Mean squares**	***F***	***P***
Year	1	3876.042	3876.042	18.99	<0.0001
Season	2	14627.620	7313.810	35.83	<0.0001
Time period	3	6030.125	2010.042	9.85	<0.0001
Types of collection	2	5916.954	2958.477	14.49	<0.0001
Season x time period	6	3662.861	610.477	2.99	0.0083
Season x collection methods	4	12326.991	3081.747	15.10	<0.0001
Time period x collection methods	6	1529.639	254.940	1.25	0.2835
Season x Time period x collection methods	12	3463.417	288.619	1.41	0.1629

Year = 1 and 2; season = dry, hot, wet; time period = early evening, late evening, pre-dawn, dawn; types of collection = indoor HLC, outdoor HLC, cow-baited net trap.

**Table 6:** **ANOVA between collection methods, mean ambient temperature, relative humidity and rainfall in Tum Sua Village, Mae Sot District, Thailand. (*P*** **< 0.05)**

**correlation**	**df**	***F* value**	**Sig. (*P*)**
Indoor HLC vs. Temp	1	1.154	0.294
Indoor HLC vs. RH	1	0.157	0.696
Indoor HLC vs. Rainfall	1	4.196	0.053
Outdoor HLC vs. Temp	1	0.960	0.338
Outdoor HLC vs. RH	1	0.063	0.804
Outdoor HLC vs. Rainfall	1	1.265	0.273
CBC vs. Temp	1	1.220	0.281
CBC vs. RH	1	0.971	0.335
CBC vs. Rainfall	1	1.908	0.181

Indoor HLC: Indoor human-landing collection.

Outdoor HLC: Outdoor human-landing collection.

CBC: Cow-baited collections.

The multiplex AS-PCR assay was used to confirm Minimus Complex species identity. Only 2 species, *An. minimus* and *Anopheles aconitus*, were identified from the collections. A total of 3,859 Minimus Complex species were collected from indoor HLC (1,197:31.02%), outdoor HLC (1,786: 46.28%) and cow baited collections (876: 22.7%). *Anopheles minimus* s.s. represented nearly all (99.8%) of the Minimus Complex members collected (n = 3,854) with only 5 (0.17%) identified as *An. aconitus*. PCR amplified species-specific products of 310 bp and180 bp can clearly separate *An. minimus* from *An. harrisoni*, respectively. *Anopheles harrisoni* was not detected during the entire study period. A total of 2,983 *An. minimus* were collected from indoor and outdoor HLC representing 24 all-night collections using 4 collectors. The total mean number of *An. minimus* per collector over the entire study period was approximately 746 or a mean of 31 landing mosquitoes per person/night. The monthly mean human-landing rates per person/night for indoor and outdoor HLC is shown in Table 3. All data collected regarding *An. minimus*, monthly total indoor and outdoor HLC, mean ambient temperature and relative humidity, and total rainfall used to describe and analyze the trophic behavior and seasonal abundance over a 23-month study is presented in Table 4.

Overall, *An. minimus* demonstrated a slight preference to feed outdoors compared with indoors and fed preferentially on humans compared to cows as offered. Fewer numbers of mosquitoes were captured from the CBC method (n = 876) than combined indoor/outdoor HLC (n = 2,983), although the mean number of mosquitoes captured per person was approximately 746 over the entire study. However, adjusting the HLC numbers to reflect that only 45 min each hour was spent collecting landing mosquitoes, the mean number of *An. minimus* per person was estimated by dividing the mean number by 0.75 to arrive at 994 mosquitoes per person. In the first year, indoor collections peaked in November, whereas two distinct peaks in outdoor activity were observed in November and May that same year (Table 3). With the highest rainfall during the entire study period occurring in July (482.5 mm), *An. minimus* was absent from all human outdoor collections and very low for indoor HLC and outdoor CBC. As the wet season continued, September recorded the lowest total of *An. minimus* for the entire study. During the second year, total outdoor collections of *An. minimus* exceeded indoor collections (*P* > 0.05). Both indoor and outdoor collections peaked in May before the onset of the wet season (Table 3).

**Figure 1:**
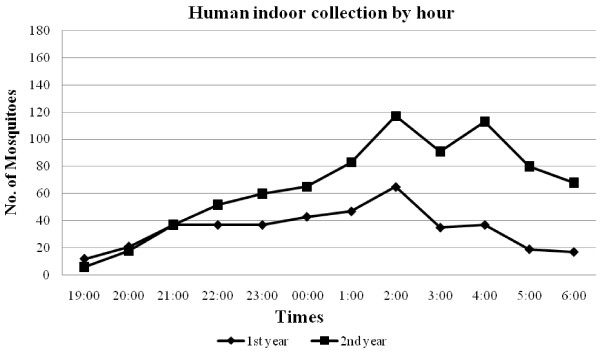
**Hourly indoor frequency of human-landing collections of*Anopheles minimus.***

**Figure 2:**
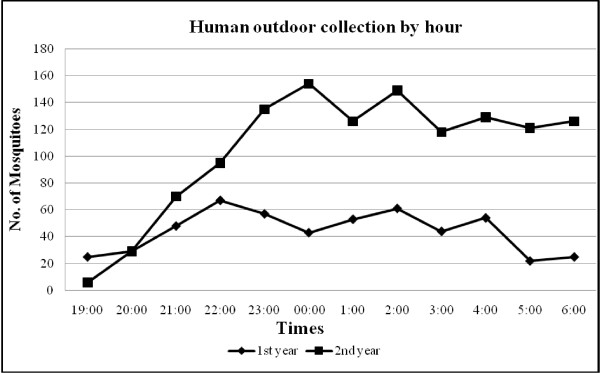
**Hourly outdoor frequency of human-landing collections of*Anopheles minimus.***

**Figure 3:**
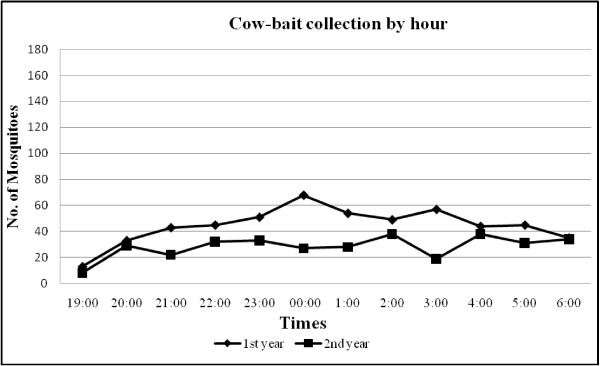
**Hourly frequency of landing activity from cow-bait collections of*Anopheles minimus.***

**Figure 4:**
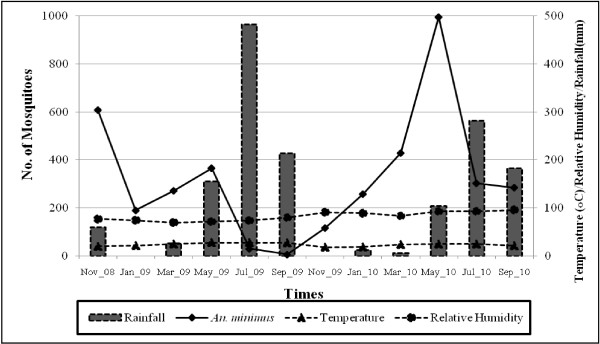
**Monthly collection of*Anopheles minimus*in relation to average monthly ambient air temperatures, relative humidity and rainfall in Tum Sua Village, Mae Sot District, Tak Province, western Thailand.**

The pattern of mean ‘feeding’ frequency of *An. minimus* by hour and method of collection are shown in Figures 1, 2, 3. Indoor activity patterns were relatively similar for both years (Figure 1), with greater activity occurring in the second half of the evening with varying points of peak attack. With outdoor HLC, increased activity began earlier in the evening compared to indoor biting with small peaks of activity seen from 2100–2200 and 0100–0200 hrs in the first year, and 2 higher, more notable peaks detected 2300–2400 and 0100–0200 hrs in the second year (Figure 2). For CBC, there were no demonstrable peaks seen throughout the evening although slightly more mosquitoes were caught around midnight during the first year collections, a reverse of what was recorded for outdoor HLC that same time period (Figure 3). *An. minimus* was found to be significantly more abundant in November in the first year and May in the second year (Figure 4).

Total number of landing mosquitoes per hour was analyzed by three-way ANOVA, comparing the 3 climatic seasons (wet, dry, hot), 4 time intervals (early evening, late evening, pre-dawn, and dawn) and 3 collection methods (indoor and outdoor HLC, CBC) (Table 5). There were significant differences in overall numbers of *An. minimus* captured between the first year and second year (*F* = 18.99, df = 1, *P* < 0.0001), between seasons (*F* = 35.83, df = 2, *P* < 0.0001), between the 3-hr time periods (*F* = 9.85, df = 3, *P* < 0.0001) and between human and cow-baited collection methods (*F* = 14.49, df = 2, *P* < 0.0001). Analysis revealed a positive association between season and time intervals of collections (*F* = 2.99, df = 6, *P* = 0.0083) and between seasons and collection methods (*F* = 15.10, df = 4, *P* < 0.0001). There was no apparent relationship between time periods and collection method alone (*F* = 1.25, df = 6, *P* = 0.2835) or between season, time period and collection method (*F* = 1.41, df = 12, *P* = 0.1629).

No significant correlation was found between the collection procedures when using either human bait as attractant (indoor or outdoor) or the cow-bait capture method (Table 6). Likewise, there was no correlation (r = 0) found between paired collection method and environmental parameters, i.e., indoor HLC vs. temperature (*P* = 0.294), indoor vs. relative humidity (*P* = 0.696), indoor vs. rainfall (*P* = 0.053), outdoor vs. temperature (*P* = 0.338) outdoor vs. relative humidity (*P* = 0.804), outdoor vs. rainfall (*P* = 0.273), cow-bait vs. temperature (*P* = 0.281), cow bait vs. relative humidity (*P* = 0.335), and cow bait vs. rainfall (*P* = 0.181) (Table 6).

## Discussion

The major objectives of the study were vector species identification, description of species diversity and abundance, and host preference/feeding behavior as background and basis for more detailed investigations to follow at the study site. Both morphological and molecular methods of anopheline species identification were used to confirm and attribute observed behavior to particular species, the focus being *An. minimus*. This knowledge is fundamental to understanding the epidemiological importance of vector species and therefore properly targeting protection methods and vector control strategies. At Tum Sua Village, the observations on the adult landing (biting) activity and host preference of *An. minimus* females covered a 23-month period of periodic sampling. Our findings showed that *An. minimus* has a more anthropophilic tendency as relatively more mosquitoes were caught on human-bait, similar in number both inside and outside of houses, than on a cow located outdoors. This study also demonstrated the impact of increased rainfall, showing a dramatic decrease in adult population numbers during the wet season compared to the dry and hot climatic periods of the year. In general, the greater the propensity to feed on humans compared to other animals is more conducive for efficient and stable malaria transmission. Because *An. minimus* was the predominant and most consistent anopheline collected at this site, malaria transmission in this area is most likely the consequence of this species. This is consistent with previous studies reporting that *An. minimus* complex play a dominant role in malaria transmission in this area of western Thailand as a consequence of relative year-round abundance and characteristics promoting efficient vectorial capacity [5,10,24,28,29].

*An. minimus* is one of the main malaria vectors on the Southeast Asian mainland [21,30-32]. This species is found from northern India eastwards through Vietnam and across southern China, Laos and Cambodia [7,17,33]. The *An. minimus* complex represents major malaria vectors in tropical and subtropical regions of China [6,24,34]. In northern Vietnam, this species has been shown to differ in some typical behavioral traits associated with the species elsewhere that carry important ramifications regarding malaria transmission and for application of vector control [32]. Whereas *An. minimus* is considered to be an important malaria vector in the Minimus Complex, the role of *An. harrisoni* remains unclear [11], thus the ability to clearly differentiate these 2 sibling species is critical in the interpretation of data and understanding of the role each contributes in the epidemiology of disease. Our ability to clearly distinguish these two species indicated only *An. minimus* was present during the study period. Without accurate species identification, it is extremely difficult to attribute vector capacity and transmission risk to a particular species and further complicates the need and design of target-specific prevention and control measures.

*Anopheles minimus* complex was the most commonly captured species throughout the study, regardless of collection method and locations, comprising 50.36% of the total anopheline species collected. Among those in the complex, 99.87% were identified as *An. minimus* with no evidence of *An. harrisoni* presence at any time during the study. This observation is consistent with previous findings from the same locality based on adult morphological identification only [20,35,36]. These findings also closely correspond to a larval distribution study by Kongmee *et al.*[37] conducted in the same village. Although a number of studies have reported on female host preference, mosquito density, biting frequency, and behavioral responses to chemicals of *An. minimus* s.l.*,* in only a few exceptions have species-specific identifications been verifiable and confirmed by molecular methods. This is important as many malaria vectors, including *An. minimus* s.l. display a diverse array (plasticity) of host-seeking behaviors, preferences, and larval breeding habitats than commonly assumed [28,29,38]. For example, Harbach *et al.*[20] observed a single feeding peak for *An. minimus* s.l., between 2100 and 2200 hr, whereas, Ratanatham *et al.*[21] reported 2 clear and consistent feeding peaks in biting density, one in the early evening (1900–2200 hr) followed by a second peak before dawn (0500–0600 hr). Rattanarithikul *et al.*[22] reported two prolonged biting periods, the first occurring between 1800 and 2300 hrs and a second, more moderate peak beginning at 0100 hr with a progressive decline in activity throughout the second half of the night.

In recent years, a molecular identification assay has been used to accurately describe the trophic behavior and biting activity of two sibling species within the Minimus Complex from Kanchanaburi Province. Unfortunately, the number of *An. minimus* obtained by Sungvornyothin *et al.*[11] was too low to observe clear biting activity patterns. In our study, indoor human landing by *An. minimus* were pronounced in the early morning hours between 0100 and 0400 hrs with a peak at 0200 hr, whereas an outdoor feeding surge began around 2200 hr, reaching a peak near midnight followed by a gradual decline throughout the second half of the evening. *Anopheles minimus* showed a slight predilection to feed more outdoors than indoors, indicating both exo- and endophagic behaviors. Additionally, a greater number of *An. minimus* were collected from human bait (indoor and outdoor) compared to cow bait, suggesting a more marked anthropophilic behavior by this species.

A significantly greater number of *An. minimus* were collected during the second year of observation compared to the first. The reasons for this difference in adult densities is unclear but is likely related to the differences in local environmental and climatic factors between collection periods and/or by the occurrence of more suitable and productive breeding habitats during the second year. In this study, rainfall appeared to have a profound influence on adult vector densities. This was very evident in the first half of the study where the lowest HLC numbers occurred during the wet months of the year (June-October) but regained adult densities immediately following the rains and into the drier and warmer periods of the second half. During the wet months of the first half of the study, particularly heavy rains occurred resulting in a 51 percent higher precipitation (1,386.7 versus 920.2 mm) than the same period of the second half of the study. As the typical larval habitats of *An. minimus* are associated with small running streams, the heavy and more frequent rainfall would have produced more adverse conditions due to flushing. In addition, higher mean relative humidity was also observed in the second year that may have favored increased vector longevity and thus greater likelihood of human-vector contact over time. A notable reduction in organized vector control measures in the area may also have allowed vector densities to increase more easily. Recently, the Thai Government has encouraged the local community to convert their land to planting rubber trees for greater income generation and may have influenced the population density of *An. minimus*. For example, *An. minimus* populations have decreased significantly in peninsular/southern Thailand following extensive ecological/environmental changes resulting from deforestation and increased urbanization [39]. Recently, *An. minimus* at Pu Teuy Village in Kanchanaburi Province has nearly disappeared, possibly the result of rapid environmental changes and increased agricultural development in the area [11].

This study had several limitations. It was difficult to measure with accuracy the host preference of the population, as the methods used may not have been as comparable as desired. For instance, were four humans the near equivalent of one cow? We presume that the specimens collected from the cow trap represented a full hour of attracted mosquitoes despite the actual collection time by the collectors was only 15 min each hour. Once the mosquitoes entered inside the net, they most likely remained inside regardless of whether or not they blood fed. On the other hand, the HLC took place for only 45 min per hour, thus we adjusted the number of actual landing mosquitoes to reflect the potential mosquitoes missed during those 15 min breaks each hour (assuming a relative even distribution of landing activity over the entire hour). Secondly, no effort was made to sample surrounding larval habitats concurrently with adult collections in attempt to link larval presence with adult densities. Thirdly, environmental measures (indoor and outdoor air temperature and RH) were routinely recorded each collection period; however, rainfall data was gathered at a station approximately 18 km away from the study site. Although not ideal because of the possibility of varying and patchy rainfall distribution patterns, we believe the relative amount of rainfall per period is still reflective of the relative amounts of rainfall and general seasonality at the study site. Lastly, this study was not an all-inclusive investigation about the vector biology of this species; neither age-grading, (parity) nor determination of malaria infection in *An. minimus* was examined as part of the baseline design. Therefore, we have no information of the change in age structure over the 23-month period or the number of mosquitoes that may have been harboring malaria parasites. Parity (age-grading) has been reported previously for *An. minimus* elsewhere in Thailand [22,40-42]. Despite the above reservations, the study provided clear evidence of the public health importance of this species. Coupled with numerous instances of confirmed vector involvement in western Thailand, our findings reinforce the conclusion regards the primary vector status of *An. minimus* in Tum Sua Village.

The Communicable Disease Control section of the MOPH has described Tum Sua Village as a perennial transmission area, albeit one showing seasonal fluctuations and with generally low transmission most of the year. *Anopheles minimus* has been regarded as the main malaria vector in this area for many decades [5,10]. Our study supports that conclusion based on biting behavior and host preferences compared to other potential vector species present. Vector control using residual application of insecticides remains on the forefront in the fight against malaria at Tum Sua Village. Since 1994, deltamethrin has been the primary insecticide of choice in the National Malaria Control Program [43]. Despite the many years of IRS in the village, the response of *An. minimus* to deltamethrin in Tum Sua is not known and will be the focus of future study.

## Conclusions

As *An. minimus* has shown a propensity to feed on humans and indoors, both in substantial numbers and in proportion to outdoor feeding, these findings can help explain the apparent effectiveness and strategy of using IRS for the suppression of malaria transmission indoors by this species. This would also support use of long-lasting insecticide-treated materials to protect people from malaria inside the household. These observations also indicate a high ‘residual’ transmission potential outdoors where direct control measures are lacking or insufficient. We conclude that the study of vector population behavior is crucial and highly beneficial for a better understanding of the epidemiological factors associated with promoting malaria transmission in a specific setting and in selecting the most appropriate vector control strategy for maximum cost-benefit.

## Competing interests

The authors declare that they have no competing interest.

## Authors’ contributions

All the authors have contributed significantly to this study. TC and MJB contributed to the conceptualization and design of the study. RT, CT and TC did the laboratory and field studies. RT and WJ carry out the molecular genetic studies. RT, MJB, ST, TC and VC prepared and revised the manuscript. All authors read and approved the final manuscript.
